# Achilles Tendon Allograft with Semitendinosus Autograft Reinforcement Is a Successful Treatment for Chronic Patellar Tendon Rupture: Report of Two Cases

**DOI:** 10.1155/2021/9951754

**Published:** 2021-08-09

**Authors:** Lucie Regennass, Mathieu Boissard, Alban Fouasson-Chailloux, Ronan Guillou, Cecile Toanen, Vincent Crenn

**Affiliations:** ^1^Orthopedics and Trauma Department, University Hospital of Nantes, Nantes, France; ^2^Orthopedics and Trauma Department, Polyclinique de l'Atlantique, Nantes, France; ^3^Physical Medicine and Rehabilitation Center, University Hospital of Nantes, Nantes, France; ^4^Orthopedics and Trauma Department, Nouvelles Cliniques Nantaises, Nantes, France; ^5^Orthopedics and Trauma Department, Departmental Hospital Center of La Roche-sur-Yon, La Roche-sur-Yon, France

## Abstract

Rupture of the patellar tendon must be diagnosed urgently because reconstruction of the extensor mechanism produces better results when it is performed in acute conditions. Reconstruction of chronic extensor mechanism rupture on the contrary is very challenging. Several surgical techniques have been described using a variety of graft choices and fixation methods, but the optimal approach is still under debate. We report our experience of two cases of chronic patellar tendon rupture reconstruction using an Achilles tendon allograft reinforced by a vascularized ipsilateral semitendinosus tendon frame. The rapid functional recovery of the range of motion, only three months postoperatively, showed us that this reconstruction technique was effective.

## 1. Introduction

Chronic patellar tendon rupture is an uncommon pathology that is debilitating for patients, and its treatment can present a challenge for the treating surgeon. There are various etiologies: posttraumatic, secondary to systemic inflammatory pathologies [1, 2], or even iatrogenic postprosthetic knee surgery [3]. Reconstruction of a ruptured extensor apparatus provides better outcomes when performed in the acute phase (<2 weeks) [4, 5]. This can be explained by patellar migration, quadriceps retraction and atrophy, and poor quality of the soft tissues, all of which occur when treatment is delayed.

To date, there are few studies and little data regarding the late reconstruction of a ruptured extensor apparatus [5–10]. Several approaches can be used, but all have their drawbacks. For example, reconstructions using autografts have the disadvantage of weakening the contralateral lower limb and a primary repair technique such as suture [11] reinforced by a metal or biological frame [12] showed a high risk of further rupture.

This article reports our original experience of two cases of late reconstruction of the patellar tendon using an Achilles tendon allograft with semitendinosus reinforcement.

## 2. Presentation of the Cases

### 2.1. Patient Case 1

**Figure 1:**
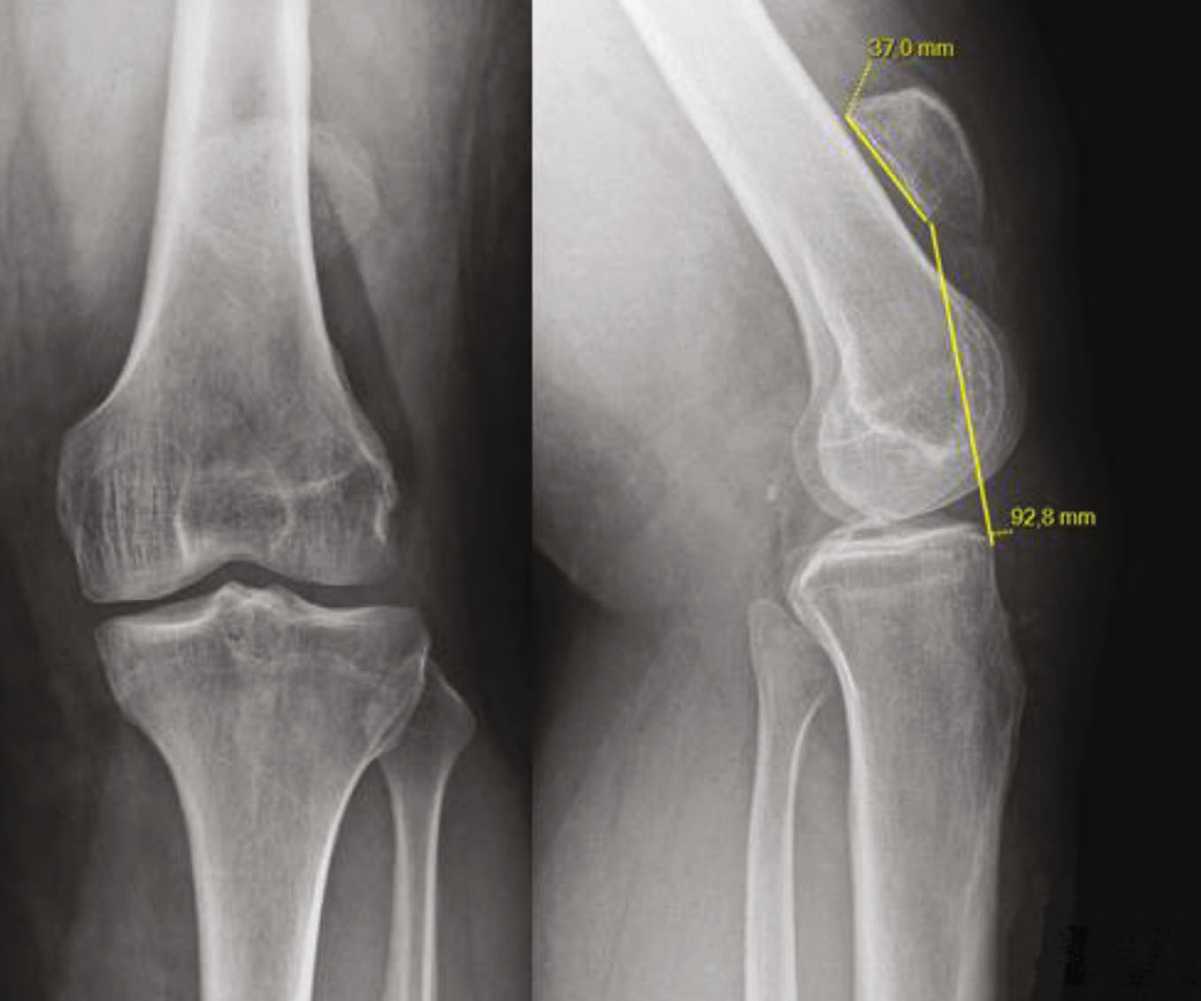
Preoperative X-rays (anteroposterior and lateral views) of patient 1: patella alta, Caton Deschamps index = 2.47.

**Figure 2:**
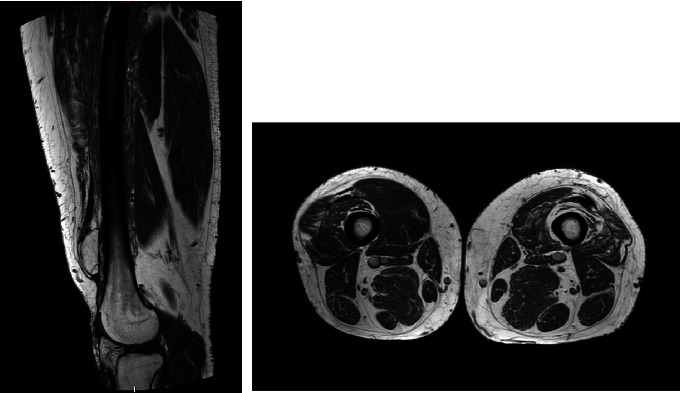
Preoperative MRI of patient 1: atrophy and poor tissue quality of the left quadriceps muscle.

Patient 1 was a 41-year-old male with a past history of a road traffic accident that resulted in a ruptured left patellar tendon. He was initially treated conservatively with splint immobilization. Our treatment took place four years after the initial trauma. The patient complained of chronic pain with no possibility of locking for full extension of his knee. The preoperative range of motion was rated at 5 degrees of flexion deformity and 130 degrees of flexion. In addition, active extension was impossible. No laxity in the frontal or sagittal plane was identified. The Caton Deschamps index measured 2.47 on the X-rays, and the patella was 9 cm from the anterior tibial tuberosity (ATT) (Figure 1). The preoperative magnetic resonance imaging (MRI) showed significant atrophy and poor tissue quality of the quadriceps muscle (Figure 2).

### 2.2. Patient Case 2

**Figure 3:**
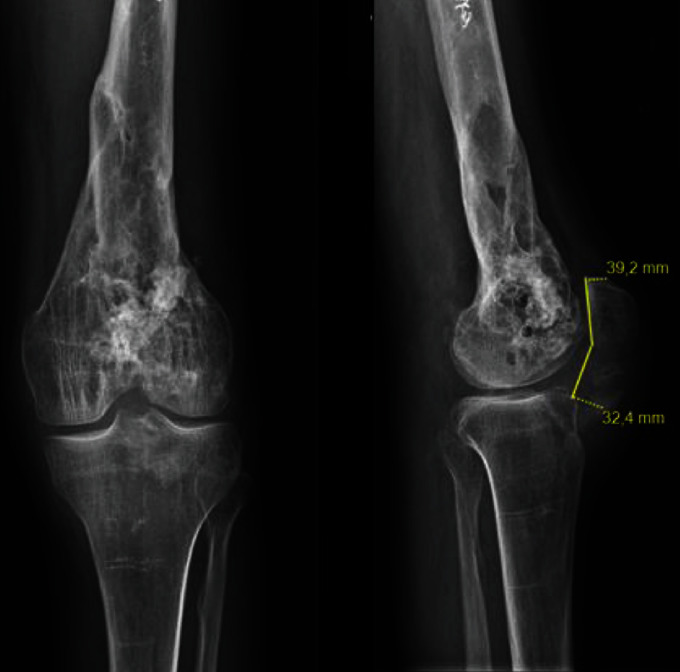
Preoperative X-rays (anteroposterior and lateral views) of patient 2. Caton Deschamps index = 0.82.

Patient 2 was a 45-year-old male with a history of multiple injuries to his left knee. A road accident was responsible for a supra- and intercondylar fracture of the femur, managed with osteosynthesis and a supracondylar nail. The nail was removed two years later because of a conflict between the femoral nail and the patella. Secondary to this, he presented with sepsis requiring surgical revision for washing, and he was treated with antibiotic therapy. Subsequently, X-rays showed calcifications in the patellar tendon leading to knee stiffness. Following a fall, an intratendinous calcification fracture was responsible for rupture of the extensor mechanism. Our treatment came two months after the trauma. The range of motion was graded as no flexion deformity, no recurvatum, and 30 degrees of flexion, and active extension was impossible. The Caton Deschamps index measured 0.82 on the X-rays (Figure 3).

We decided to reconstruct their extensor apparatus using an Achilles tendon allograft reinforced by a semitendinosus frame. Both patients were informed that their history could be used to produce a scientific article. They provided their consent. Preoperatively, the range of motion was measured using a goniometer. The visual analog scale measured their pain. Our imaging workup included bilateral AP and lateral X-rays of the knees. The Caton Deschamps index [13] was used to measure the height of the patella. X-rays of the healthy side were used to assess the standard height of the patella. A reconstruction that was too long would compromise the benefit of reconstructing the extensor mechanism, while one that was too short would lead to limited flexion and a risk of rerupture. However, the patients were also warned preoperatively that regular, symmetrical restoration of the patellar tendon length was unlikely.

### 2.3. Operating Technique

The patients were positioned in the supine position with a knee-bar at 60 degrees of knee flexion. An air tourniquet was applied to the base of the limb at a pressure of 250 mmHg. General anesthesia was used for complete muscle relaxation, associated with a femoral nerve block. Antibiotic prophylaxis was administered. We used an anterior medial approach.

Preparation of the receiver site consisted of making a four-sided hole in the ATT: width 2 cm, height 4 cm, and depth 2 cm. A bone block that is too loose or too small can lead to increased risk of nonunion. We then created a patellar trench: width 1.5 cm and depth 1 cm, from the upper pole to the lower pole, on the anterior cortical.

We then prepared the Achilles tendon allograft by cutting the calcaneal block to fit the trench size created in the ATT.

Tibial fixation of the allograft was performed using two 4.5 mm metallic screws in the calcaneum block and two 18 mm wide U-type staples stabilizing the calcaneum block on the medial and lateral sides of the ATT. The Achilles tendon graft needs to be fixed in extension after the optimal patellar height is achieved by tensioning, under fluoroscopic visualization, while the knee is at 30 degrees of flexion. In order to obtain the optimal patellar height, patient 1 needed the quadriceps to be released; patient 2 did not. The quadriceps tendon was released by using a Cobb on the anterior cortical area of the femur and severing the medial and lateral patellar retinaculum. Patellar fixation was achieved by placing the tendinous part of the allograft in the patellar trench and securing it with transosseous stitches with FiberWire® Size 2 (Arthrex).

**Figure 4:**
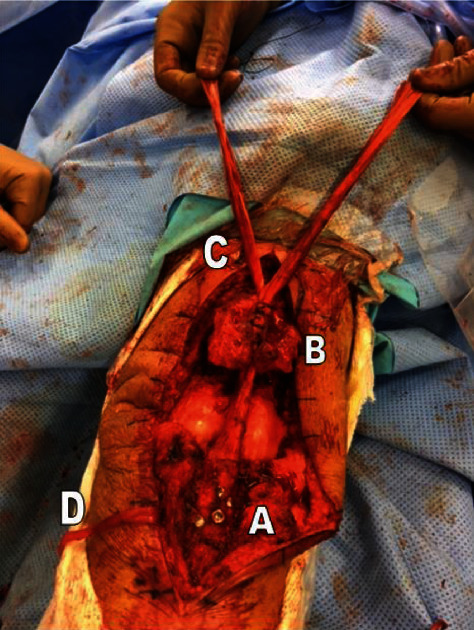
Fixation of the calcaneal block on the ATT using two screws and two staples (A). Fixation of the Achilles tendon allograft in the patellar trench (B) and division of the rest of the tendon into two strands (C). Semitendinosus tendon harvesting (D).

Once the allograft was attached to the tibia and patella, we split the excess tendon in the patella's upper pole into two strands (Figure 4). These strands were passed through the vastus medialis and vastus lateralis and then resutured onto each other and around the prepatellar rim.

**Figure 5:**
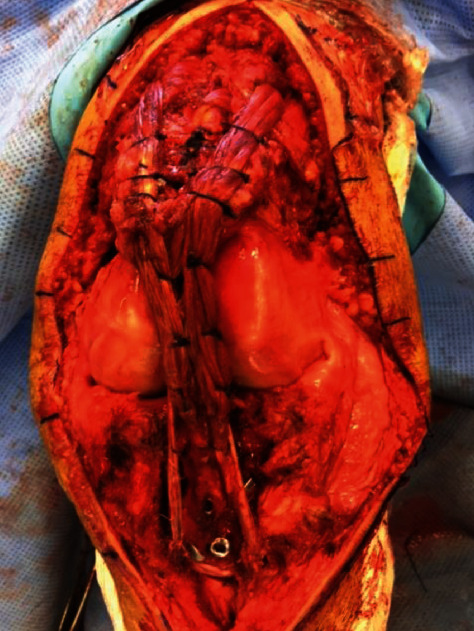
Framing with the semitendinosus tendon. Suture of the two strands onto each other and around the patella after passing through the vastus medialis and vastus lateralis.

Autologous homolateral semitendinosus was harvested, keeping its distal insertion to make a vascularized graft possible. It was fixed on both sides of the allograft with 0 absorbable stitches (Figure 5), and the knee flexed at 60 degrees.

Both our patients were treated postoperatively with preventive antibiotic therapy while awaiting the various bacteriological results from samples taken from both the graft and the patients themselves. The results of the complete bacteriological analysis were returned at 2 weeks; these samples were sterile on day 15, making it possible to stop the antibiotic therapy.

Weight-bearing was authorized from day 1 with an extension splint. The splint was kept for 6 weeks. Rehabilitation started on day 1 and consisted of awakening the quadriceps and recovering mobility. Flexion was only allowed up to 30 degrees in the first three weeks.

## 3. Results

**Figure 6:**
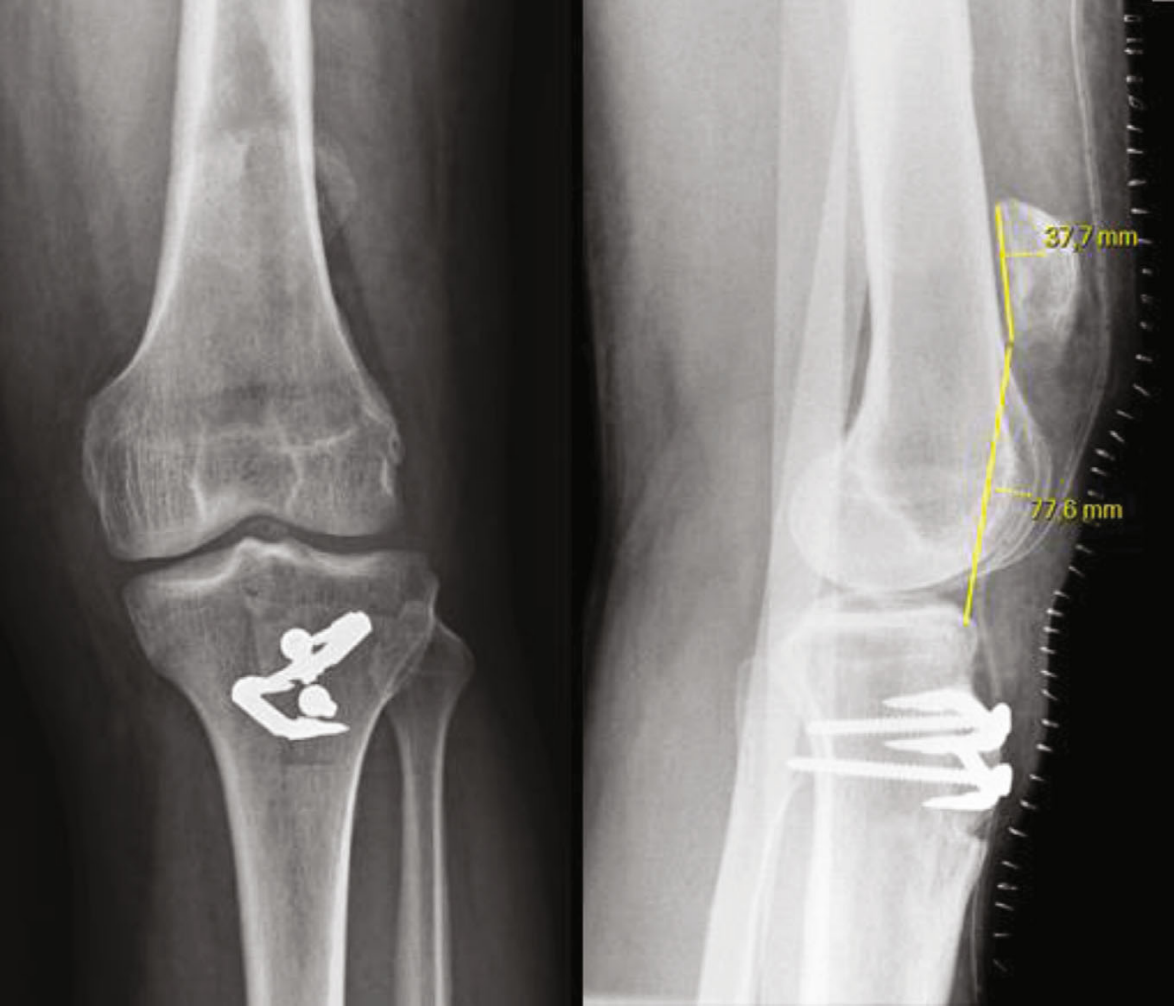
Postoperative X-rays (anteroposterior and lateral views) for patient 1: Caton Deschamps index = 2.

**Figure 7:**
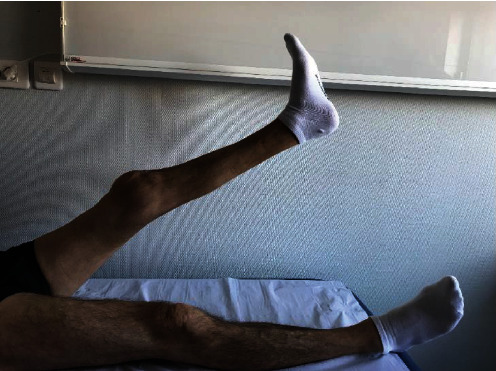
Active extension and locking.

For patient 1, the consultation four weeks after surgery revealed quadriceps contraction, but lock-in extension had not yet been acquired. The range of motion was rated at 60 degrees of flexion without flexion deformity or recurvatum. On the postoperative X-rays, the Caton Deschamps index was calculated at 2 (versus 2.47 preoperatively), and no material fixation failure was observed (Figure 6). Two months after surgery, knee locking was acquired, and the range of motion was rated at 85 degrees of flexion with neither flexion deformity nor recurvatum (Figure 7). At one year, the patient had no pain; in particular, no patellar syndrome was observed, driving was possible, and his walking range was unlimited. The range of motion was 120 degrees of flexion with neither flexion deformity nor recurvatum. In contrast, the weakened quadriceps muscle persisted.

We performed isokinetic strength evaluation using a dynamometer (Medimex, France), 18 months after surgery. The aim of isokinetic tests is to assess the strength of a muscle group in a dynamic way, by getting as close as possible to physiological work. Torque was gravity-corrected to 45 degrees of knee flexion. The knees were evaluated, beginning with the nonoperated side after providing instructions and with both verbal encouragements and visual feedback. After familiarization with the isokinetic movement (5 concentric submaximal repetitions at 240°/s), the patient was tested over 3 repetitions in the concentric mode at 60°/s followed by 5 concentric repetitions at 180°/s. Thirty seconds of rest was provided between the two series and 2 minutes between the two sides. The concentric strength peaks of the knee extensors assessed at the two angular velocities of 60 and 180 degrees per second were used as study parameters to calculate the quadriceps limb symmetry index using the following formula: (peak torque of the operated side/peak torque of the nonoperated side) × 100.

This test showed a significant deficit of 90% in left quadriceps strength (right quadriceps at 60°/sec: 142 Newton meters and at 180°/sec: 99 Newton meters vs. the left knee at 60°/sec and 180°/sec: 16 Newton meters for both).

**Figure 8:**
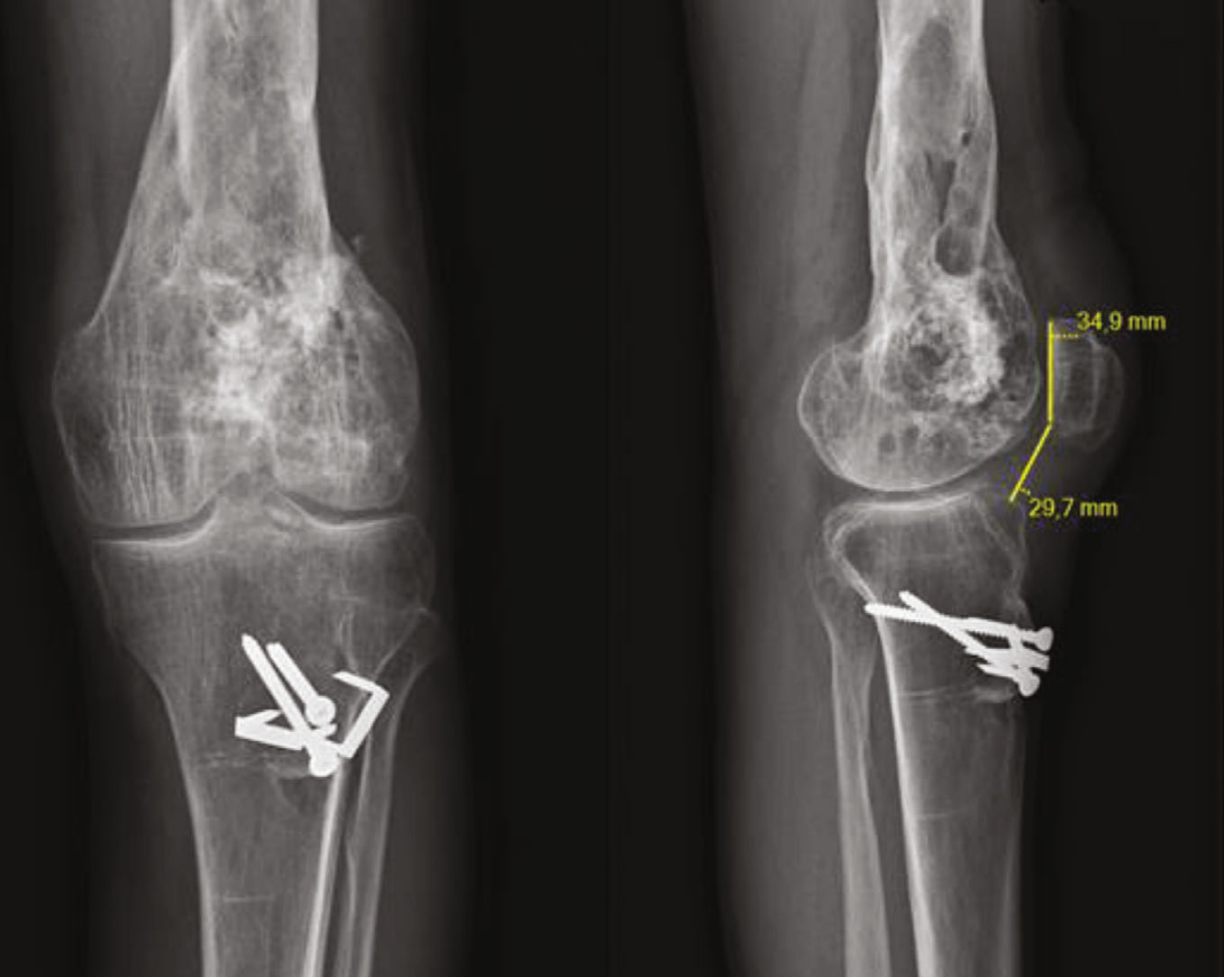
Postoperative X-rays of patient 2: Caton Deschamps index = 0.85.

For patient 2, at one month, knee locking was already acquired and there was no pain. Ranges of motion were rated at 50 degrees of flexion without flexion deformity or recurvatum. Motor testing of the quadriceps was 3/5. On the postoperative X-rays, the Caton Deschamps index was calculated at 0.85 (versus 0.82 preoperatively); no material fixation failure was observed (Figure 8). At three months, the range of motion was 80 degrees of flexion with neither flexion deformity nor recurvatum, and at 1 year, his flexion was 90 degrees. Unfortunately, the patient refused to do isokinetic measurements one year after surgery.

Finally, there were no skin or septic complications in either of our patients.

## 4. Discussion

After chronic patellar tendon rupture, reconstruction of the extensor mechanism is an unusual and challenging surgical approach because of tissue retraction, patellar migration, quadriceps atrophy, and poor tissue quality. In a review article on extensor mechanism injuries, Pengas et al. [14] reported unfavorable results for chronic injuries of the patellar tendon, especially those in which the patella was retracted by the quadriceps to a proximal position. Several techniques have been described for restoring the extensor mechanism: reconstruction using autologous hamstrings [6], a metal frame, Z-plasty of the patellar or quadriceps tendon, use of an autograft of the fascia lata, or even polyethylene tape as an artificial ligament [7]. To date, there are few studies on Achilles tendon allograft reconstruction [2, 3, 8, 12–15].

The advantage of an Achilles tendon allogeneic transplant is that it provides the graft with good tissue and bone quality in patients whose tissues are particularly fragile. Lamberti et al. demonstrated in their recent article that allografts in general, and Achilles tendon allografts in particular, provided better functional results than autografts in restoring correct joint function following total knee arthroplasty [16]. They also reported good results by performing an ipsilateral quadriceps and semitendinosus autograft using the Rajgopal et al. technique [17]. However, in our case, the bad quality of the tissues did not allow us to perform an ipsilateral autograft. That is why performing a preoperative MRI in order to characterize the lesions is important in the therapeutic decision.

Our technique also has the advantage of using several fixation methods: an Achilles allograft, a hamstring autograft, and FiberWire®.

Bone fixation was achieved using screws and staples, to which we added an autograft of cancellous bone. Under these conditions, we believe that this fixation provides good primary stability and may improve bone integration. Fixation of the tendinous part of the allograft to the patella, through the vastus medialis and vastus lateralis, is an essential part of our surgery. We believe that this makes possible better distribution of the forces and decreases the stress on the quadriceps muscle, the tissues of which are already damaged.

The advantage of our technique is also that there was framing of the semitendinosus. Adding this biological fixation made it possible to strengthen our fixation while avoiding the need for another surgery to remove the material used for the metallic framing. This biological framing should reinforce the reconstruction's long-term stability, bearing in mind the risk of allograft resorption in the long term. As we can see in the case reported by Ginesin et al. [18], the hamstring has many advantages: it protects the allograft reconstruction thanks to its distal attachment, in addition to being a vascularized graft. McNally [15] also used a hamstring autograft for patellar tendon reconstruction and described a system with two adjustable cortical buttons, thinking that the adjustable suspensory fixation would help distribute the tension and determine the patellar height needed for a functioning knee joint.

In our study, the functional results were excellent. Both our patients recovered active extension at three months after surgery. Walking with technical assistance and immobilization with a splint was thus needed for less than two months. The range of motion in their knees is compatible with everyday activities from 0 degree to more than 90 degrees, and patellar alignment has been restored and maintained.

The main disadvantages of our technique are the risk of infection associated with using an allograft, the risk of patellar fracture, and the cost and availability of the allograft. Nevertheless, neither of our patients experienced any complications.

In patient 1, we failed to restore height to the patella. Studies have reported the benefits of continuous preoperative transpatellar traction in the bed plane [4, 19]. However, this technique requires an extended hospital stay and increases the risk of infection. In addition, the results after traction are random.

In our study, we were confronted with two different cases because patient 1 presented with a patella alta, while patient 2 had a normal patellar height. Our primary goal was to restore continuity in the extensor mechanism, with good function. This is why we were not expecting to fully restore the patella height, and this was not our goal.

We identified quadriceps atrophy, and quadriceps muscle weakness persisted. This is consistent with the cases reported by Falconiero and Pallis [8] and Pengas et al. [14]. However, our two patients recovered active extension and joint mobility compatible with everyday activities. We believe that extended rehabilitation might improve the muscular strength of the quadriceps.

Regarding the range of motion, while the second patient's joint mobility was only 90 degrees of flexion at one year, it should be remembered that his preoperative mobility was only 30 degrees.

## Data Availability

The data that support the findings of this study are available from the corresponding author, LR, upon reasonable request.
